# A multicenter, double-blind, randomized, parallel-group, placebo-controlled study to evaluate the efficacy and safety of camostat mesilate in patients with COVID-19 (CANDLE study)

**DOI:** 10.1186/s12916-022-02518-7

**Published:** 2022-09-27

**Authors:** Taku Kinoshita, Masahiro Shinoda, Yasuhiro Nishizaki, Katsuya Shiraki, Yuji Hirai, Yoshiko Kichikawa, Kenji Tsushima, Masaharu Shinkai, Naoyuki Komura, Kazuo Yoshida, Yasutoshi Kido, Hiroshi Kakeya, Naoto Uemura, Junichi Kadota

**Affiliations:** 1 Department of Pulmonary Medicine, International University of Health and Welfare Narita Hospital, Narita, Japan; 2 Present Address: Respiratory Medicine, Chiba Rosai Hospital, Chiba, Japan; 3 Department of Respiratory Medicine, Tokyo Shinagawa Hospital, Tokyo, Japan; 4 Tokai University Tokyo Hospital, Tokyo, Japan; 5 Department of General and Laboratory Medicine, Mie Prefectural General Medical Center, Yokkaichi, Japan; 6 Department of Infectious Diseases, Tokyo Medical University Hachioji Medical Center, Hachioji, Japan; 7 Department of Respiratory Medicine, Mishuku Hospital, Tokyo, Japan; 8 Clinical Development Planning, Ono Pharmaceutical Co., Ltd., Osaka, Japan; 9 Department of Statistical Analysis, Ono Pharmaceutical Co., Ltd., Osaka, Japan; 10 Department of Parasitology and Research Center for Infectious Disease Sciences, Graduate School of Medicine, Osaka City University, Osaka, Japan; 11 Present Address: Department of Virology and Parasitology, Graduate School of Medicine, Osaka Metropolitan University, Osaka, Japan; 12 Present Address: Research Center for Infectious Disease Sciences, Graduate School of Medicine, Osaka Metropolitan University, Osaka, Japan; 13 Department of Infection Control Science, Graduate School of Medicine, Osaka City University, Osaka, Japan; 14 Present Address: Department of Infection Control Science, Graduate School of Medicine, Osaka Metropolitan University, Osaka, Japan; 15 Department of Clinical Pharmacology and Therapeutics, Faculty of Medicine, Oita University, 1-1 Idaigaoka, Hasama-machi, Yufu-shi, Oita-ken, 879-5593 Japan; 16 Department of Respiratory Medicine and Infectious Diseases, Faculty of Medicine, Oita University, Oita, Japan; 17 Nagasaki Harbor Medical Center, Nagasaki, Japan

**Keywords:** Camostat mesilate, COVID-19, Randomized controlled trial, SARS-CoV-2

## Abstract

**Background:**

In vitro drug screening studies have indicated that camostat mesilate (FOY-305) may prevent SARS-CoV-2 infection into human airway epithelial cells. This study was conducted to investigate whether camostat mesilate is an effective treatment for SARS-CoV-2 infection (COVID-19).

**Methods:**

This was a multicenter, double-blind, randomized, parallel-group, placebo-controlled study. Patients were enrolled if they were admitted to a hospital within 5 days of onset of COVID-19 symptoms or within 5 days of a positive test for asymptomatic patients. Severe cases (e.g., those requiring oxygenation/ventilation) were excluded. Patients were enrolled, randomized, and allocated to each group using an interactive web response system. Randomization was performed using a minimization method with the factors medical institution, age, and underlying diseases (chronic respiratory disease, chronic kidney disease, diabetes mellitus, hypertension, cardiovascular diseases, and obesity). The patients, investigators/subinvestigators, study coordinators, and other study personnel were blinded throughout the study. Patients were administered camostat mesilate (600 mg qid; four to eight times higher than the clinical doses in Japan) or placebo for up to 14 days. The primary efficacy endpoint was the time to the first two consecutive negative tests for SARS-CoV-2.

**Results:**

One-hundred fifty-five patients were randomized to receive camostat mesilate (*n* = 78) or placebo (*n* = 77). The median time to the first test was 11.0 days (95% confidence interval [CI]: 9.0–12.0) in the camostat mesilate group and 11.0 days (95% CI: 10.0–13.0) in the placebo group. Conversion to negative viral status by day 14 was observed in 45 of 74 patients (60.8%) in the camostat mesilate group and 47 of 74 patients (63.5%) in the placebo group. The primary (Bayesian) and secondary (frequentist) analyses found no significant differences in the primary endpoint between the two groups. No additional safety concerns beyond those already known for camostat mesilate were identified.

**Conclusions:**

Camostat mesilate did not substantially reduce the time to viral clearance, based on upper airway viral loads, compared with placebo for treating patients with mild to moderate SARS-CoV-2 infection with or without symptoms.

**Trial registration:**

ClinicalTrials.gov, NCT04657497. Japan Registry for Clinical Trials, jRCT2031200198.

**Supplementary Information:**

The online version contains supplementary material available at 10.1186/s12916-022-02518-7.

## Background

SARS-CoV-2 is a highly transmissible virus that causes a potentially severe infection (COVID-19), which is continuing to spread worldwide and thus represents a significant global health threat [1, 2]. The symptoms and severity of COVID-19 vary considerably. Some patients develop advanced disease within about 10 days of onset, with life-threatening symptoms, including severe inflammatory reactions, dyspnea, and severe acute pneumonia [3, 4]. Another challenge is the emergence of novel variants displaying altered transmissibility, infectiveness, disease severity, and mortality risk.

Currently, severe cases are generally treated with remdesivir, dexamethasone, and symptomatic therapies [5]. However, appropriate treatments have not been well established for asymptomatic patients or patients with moderate symptoms who do not require oxygen therapy [5]. Convenient oral drugs that can be prescribed to patients recuperating at home or other outpatient settings are also needed.

As one potential therapeutic target, it was discovered that the spike protein (S protein) of SARS-CoV-2 binds to angiotensin-converting enzyme II (ACE2) on the host cell membrane as a functional receptor [6, 7]. The S protein is then cleaved into S1 and S2 by host-derived protease activity. The S1 fragment binds to ACE2, and the S2 fragment is cleaved by a type II transmembrane serine protease (TMPRSS2) expressed on the host cell membrane. These steps promote the fusion of the viral envelope (outer membrane) with the cell membrane. Therefore, ACE2 and TMPRSS2, which are expressed on airway epithelial cells, are key factors in SARS-CoV-2 infection. In vitro drug screening studies have indicated that 4-(4-guanidinobenzoyloxy)phenylacetic acid (GBPA), the active metabolite of the serine protease inhibitor camostat mesilate (FOY-305), inhibits TMPRSS2 and prevents SARS-CoV-2 infection of a human airway epithelial cell-derived cell line (Calu-3 cells) [7–14].

Repurposing drugs that have already been approved for other indications may help facilitate the drug development process and shorten the development time [15, 16]. In Japan, camostat mesilate is an oral drug that has been used to treat the acute symptoms of chronic pancreatitis and postoperative reflux esophagitis for more than 30 years and has shown a good safety profile over this period of time [17].

Based on the preclinical evidence, it has been postulated that camostat mesilate may also be useful for treating COVID-19. In support of this hypothesis, one retrospective study of critically ill COVID-19 patients with organ failure treated in an intensive care unit revealed a decline in disease activity within 8 days of admission among patients treated with camostat mesilate but not in patients treated with hydroxychloroquine [18]. This clinical improvement was accompanied by a decline in inflammatory markers, such as C-reactive protein and interleukin-6, and increased oxygenation.

This double-blind phase 3 study was conducted in Japan to evaluate the efficacy and safety of camostat mesilate and hence explore the role of TMPRSS2 as a potential treatment target for mild to moderate SARS-CoV-2 infection with or without symptoms.

## Methods

Further information about the design of this study, including patient eligibility, is available in the English version of the study protocol (Additional file 1: Study protocol). The study was registered on ClinicalTrials.gov (NCT04657497) and the Japan Registry for Clinical Trials (jRCT2031200198).

### Ethics

This study adhered to the Declaration of Helsinki, Good Clinical Practice, and relevant local/international guidelines. The protocol and patient consent forms were approved by the ethics committees or institutional review boards at all participating institutions (Additional file 2: IRB information).

### Patients

Patients aged at least 18 years were eligible for this study if they were admitted to the participating hospitals within 5 days of onset of SARS-CoV-2 symptoms or within 5 days of a positive test for asymptomatic patients. SARS-CoV-2 infection must have been tested using a standard method at the time the study was conducted (e.g., reverse transcriptase-polymerase chain reaction [RT-PCR] test, loop-mediated isothermal amplification [LAMP] test, or antigen test). Only patients with asymptomatic/mild or moderate infection were eligible. Patients with severe infection, such as those requiring oxygenation, ventilation, or admission to an intensive care unit, were excluded. The major exclusion criteria were prior history of SARS-CoV-2 infection, history of vaccination for SARS-CoV-2, and history of treatment with camostat mesilate or nafamostat mesilate. Further eligibility criteria are described in the study protocol (Additional file 1: Study protocol). All patients provided written informed consent.

### Study design

The study comprised a double-blind phase (up to 14 days) in which patients were randomized to receive camostat mesilate or placebo and a 2-week follow-up period after the last dose of the study drug.

Randomization was performed using a minimization method with the following randomization factors: medical institution, age (at least 65 *vs* less than 65 years), and absence/presence of underlying diseases (chronic respiratory disease, chronic kidney disease, diabetes mellitus, hypertension, cardiovascular diseases, and obesity [body mass index, BMI, at least 30 kg/m^2^]). Patients were enrolled, randomized, and allocated to the appropriate treatments by the investigators/subinvestigators using an interactive web response system, which was managed by the sponsor. Patients, investigators/subinvestigators, study coordinators, and other study personnel were blinded throughout the study.

The length of the double-blind period (up to 14 days) was chosen because of the typical clinical course [19].

There were no changes to the study that were implemented after commencing enrollment.

### Interventions

Eligible patients were allocated to either camostat mesilate or placebo film-coated tablets, which were visually indistinguishable in appearance and packaging, to be administered at a dose of 600 mg four times daily (qid; before breakfast, before lunch, before dinner, and bedtime) for up to 14 days. The administration status was confirmed by the clinical study staff, such as the principal investigator. The dose of camostat mesilate was chosen based on (1) preclinical half maximal effective concentration (EC_50_) values, which determined the clinical target exposures; (2) modeling and simulation to predict high dose exposure in the clinic; and (3) results of a phase 1 study of the safety and pharmacokinetics of camostat mesilate at 600 mg qid in healthy Japanese volunteers [20].

During the study, it was prohibited to administer drugs with antiviral effects (e.g., remdesivir, favipiravir, ciclesonide, nafamostat mesilate, hydroxychloroquine, ivermectin, combination drug of lopinavir and ritonavir, povidone-iodine) and drugs with anticytokine effects (e.g., tocilizumab, Janus kinase inhibitors) from the day of onset of symptoms until completion of the study. However, these drugs could be continued at the same dose in patients already using them to treat a pre-existing comorbidity. The use of other unapproved drugs or camostat mesilate as a commercial product was prohibited.

In the randomized period, the allocated treatment was to be discontinued in accordance with the study criteria listed in Additional file 2: Table S1, which included patient request, emergence of an adverse event that made it difficult to continue the study, negative test for SARS-CoV-2 on two consecutive occasions, and increasing disease severity (exacerbation of pneumonia and SpO_2_ of 93% or less despite oxygen therapy). Efficacy evaluations were not conducted after confirmation of SARS-CoV-2 negativity. Treatments beyond day 14 were at the attending physician’s discretion or institutional policies and were not recorded.

### Endpoints

The primary efficacy endpoint was the time to the first two consecutive negative SARS-CoV-2 tests performed at the hospital’s local laboratory. The local tests were used for the primary endpoint in consideration of the time involved to send and analyze samples at the central laboratory and the potential difficulty of hospitalizing patients until the central laboratory had processed the tests.

Secondary efficacy endpoints were the time to the first two consecutive negative SARS-CoV-2 tests performed at the central laboratory, the proportion of patients testing negative for SARS-CoV-2, the ordinal scale for disease severity (Additional file 2: Table S2) [21], the proportion of patients requiring mechanical ventilation, and survival.

Exploratory efficacy endpoints were the SARS-CoV-2 viral load (measured at the central laboratory), presence of lung lesions on chest imaging, time to resolution of clinical symptoms, proportion of patients in whom the clinical symptoms resolved, and antibody responses (IgM and IgG; measured at the central laboratory).

IgM and IgG targeting the spike protein of the virus were detected using a lateral flow immunofluorescence assay kit (SARS-CoV-2 IgM and IgG Quantum Dot Immunoassay, Mokobio Biotechnology R&D, Rockville, MD, USA). The fluorescence signal was semiquantified by an immunofluorescence analyzer (Mokosensor-Q100, Mokobio Biotechnology R&D).

SARS-CoV-2 tests were performed daily throughout the treatment period at each hospital’s laboratory using the locally available methods. For the central evaluations, samples were sent to a central laboratory and analyzed using a standardized RT-PCR method.

Safety evaluations included monitoring of adverse events, laboratory tests, vital signs, and 12-lead electrocardiography throughout the study.

### Statistical analyses

The objective of this study was to establish the superiority of camostat mesilate over placebo in patients with COVID-19 using the time to negative SARS-CoV-2 test as the primary endpoint.

The criteria for effectiveness were a Bayesian posterior probability of at least 92% with a hazard ratio exceeding 1.0. The criteria for ineffectiveness were a Bayesian posterior probability of 8% or less with a hazard ratio exceeding 1.0.

The time to a negative SARS-CoV-2 test was assumed to follow an exponential distribution, with a median time of 14 days in the placebo group and a median time of 7 to 8 days in the camostat mesilate group. The probabilities of meeting the assessment of effectiveness or ineffectiveness with 50 patients per group (100 in total) were calculated at various analysis time points by applying numerical simulations in SAS version 9.4. Because the timing of the interim analysis was dependent on the status of the COVID-19 outbreak, numerical simulations were performed, assuming the time points when 40 to 80 subjects would have been randomized. The prior distribution of the regression coefficient was assumed to be uniform. In this study, the number of subjects was designed using the Bayesian approach. Based on the numerical experiment, irrespective of which time point the interim analysis was performed, the probability that treatment would be effective was 75 to 90% if the median duration was 7 or 8 days in the camostat mesilate group. Moreover, if the median duration was 14 days, the probability that treatment was effective was controlled within 10% (Additional file 2: Sample size calculation). From these data, we therefore considered it was possible to demonstrate the superiority of camostat mesilate over placebo with a sample size of 100 patients (50 patients per group).

A modified intention-to-treat analysis set was used for efficacy analyses by excluding any patients who tested negative for SARS-CoV-2 on day 1 (local laboratory tests). All analyses were performed on an as-randomized basis. The safety analysis set comprised all patients who received at least one dose of the allocated drug.

Baseline characteristics were analyzed using descriptive statistics, including numbers (proportions) of patients and summary statistics, as appropriate.

For the primary endpoint, the time to SARS-CoV-2 negativity (in days) was calculated as the date of the first (of two consecutive) SARS-CoV-2 negativity test minus the date of randomization plus one. The events and reasons for censoring patients in this analysis are defined in Additional file 2: Table S3. A Cox proportional hazards model stratified by the randomization factors (age and underlying disease) was used to determine the posterior mean hazard ratio with two-sided 95% credible intervals for the camostat mesilate group relative to the placebo group. The distribution of the regression coefficients was assumed to be uniform. In a secondary analysis, we applied the log-rank test stratified by age and underlying disease and plotted Kaplan–Meier curves for both groups to calculate the median time to SARS-CoV-2 negativity, and 95% confidence intervals were calculated using the Brookmeyer–Crowley method with double log transformation. As a sensitivity analysis, we investigated the influence of patients who tested positive for SARS-CoV-2 *after* conversion to a SARS-CoV-2-negative status. The events and reasons for censoring patients in this analysis are defined in Additional file 2: Table S4.

The proportions of patients negative for SARS-CoV-2 and the distribution of disease severity were compared between the two groups using the Mantel–Haenszel test stratified by the randomization factors. The actual values for the ordinal scale of severity were compared between the treatment groups using the proportional odds model, which included treatment group and randomization factors as factors. The median time to the resolution of clinical symptoms was estimated using the Kaplan–Meier method, and 95% confidence intervals were calculated using the Brookmeyer–Crowley method with double log transformation. Changes in viral load, antibody responses (IgG and IgM), and safety outcomes were analyzed descriptively in terms of the number and percentage of patients or summary statistics, as appropriate.

All tests were two-sided with a significance level of 5%. Because the primary analysis of the primary efficacy endpoint was based on Bayesian interim monitoring, a significance level was not applied. No adjustment for multiplicity between other endpoints or time points was made.

The protocol specified that an interim analysis should be performed only if recruitment proved difficult because of the convergence of the COVID-19 pandemic during the enrollment period. It was difficult to predict the enrollment status when planning this study or to preliminarily specify the number of events to be included in the interim analysis. The Bayesian approach allowed us to make assessments without a prespecified number of events to be included in the interim analysis. Therefore, Bayesian analysis was adapted for the primary analysis, and frequentist analysis was performed for a sensitivity analysis.

Some changes in the statistical analyses were implemented before unblinding of the data. These additional analyses were performed to further evaluate the efficacy and are summarized in Additional file 2: Table S5.

SAS version 9.4 (SAS Institute, Cary, NC, USA) was used for all statistical analyses.

## Results

### Patients

**Fig. 1:**
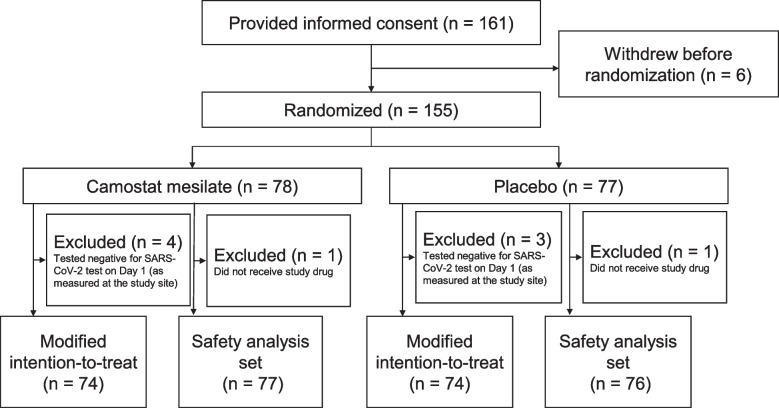
Patient disposition

Because patient enrollment progressed as planned, no interim analyses were conducted. Between November 2020 and March 2021, a total of 161 patients provided consent and 155 patients were randomized across 21 participating institutions: 78 to camostat mesilate and 77 to placebo (Fig. 1 and Additional file 2: Table S6). The study enrolled more patients than originally planned in the protocol. This clinical trial required urgent registration during the pandemic, and the number of infected patients fluctuated markedly in Japan, making it difficult to manage the completion of enrollment. The expected date of completing enrollment was announced in advance based on the rate of enrollment across 25 study sites. Patients enrolled by the time of this announcement were eligible and resulted in over-enrollment. Four patients in the camostat mesilate group and three in the placebo group were excluded from the modified intention-to-treat population due to negative SARS-CoV-2 tests on day 1; thus, the modified intention-to-treat analysis set comprised 74 patients in each group. Because one patient in each group did not receive the allocated treatment, the safety analysis set comprised 77 patients in the camostat mesilate group and 76 in the placebo group.

Among 155 patients, 78 (50.3%) were male and 77 (49.7%) were female, 59 (38.1%) were at least 65 years old, and 71 (45.8%) had at least one underlying disease, the most common being hypertension in 44 patients (28.4%) (Table 1). The median interval between the onset of symptoms (date of positive test for asymptomatic patients) and the date of registration was 4 days (range 0–5 days). RT-PCR was the predominant testing method, being used for 142 patients (91.6%). Nasopharyngeal swabs were used in 108 patients (69.7%), nasal swabs in 16 patients (10.3%), and saliva samples in 30 patients (19.4%). The median viral load was 6.91 log_10_ copies/mL (range 3.40–9.40 log_10_ copies/mL). All of the patients were hospitalized without requiring oxygen therapy (i.e., ordinal scale of 3). One hundred eight patients had symptoms at registration. Both groups were very similar, demonstrating the robustness of the randomization scheme.

**Table 1:** Patient characteristics

	**Camostat mesilate (*N* = 78)**	**Placebo (*N* = 77)**	**Total (*N* = 155)**
Sex			
Male	35 (44.9)	43 (55.8)	78 (50.3)
Female	43 (55.1)	34 (44.2)	77 (49.7)
Race			
Asian	78 (100.0)	77 (100.0)	155 (100.0)
Age at the time of consent (years)			
≥ 65	30 (38.5)	29 (37.7)	59 (38.1)
< 65	48 (61.5)	48 (62.3)	96 (61.9)
Mean ± standard deviation	55.7 ± 18.8	56.1 ± 18.2	55.9 ± 18.4
Median (range)	59.0 (21–89)	56.0 (21–94)	58.0 (21–94)
BMI (kg/m^2^)			
Mean ± standard deviation	24.5 ± 5.2	23.9 ± 3.7	24.2 ± 4.5
Median (range)	23.9 (14.1–46.2)	23.2 (18.4–33.5)	23.8 (14.1–46.2)
Underlying diseases^a^	36 (46.2)	35 (45.5)	71 (45.8)
Chronic respiratory disease	11 (14.1)	14 (18.2)	25 (16.1)
Chronic kidney disease	5 (6.4)	4 (5.2)	9 (5.8)
Diabetes mellitus	15 (19.2)	12 (15.6)	27 (17.4)
Hypertension	24 (30.8)	20 (26.0)	44 (28.4)
Cardiovascular disease	4 (5.1)	4 (5.2)	8 (5.2)
Obesity (BMI ≥ 30 kg/m^2^)	9 (11.5)	6 (7.8)	15 (9.7)
Duration from the onset date^b^ (days)			
< 4	43 (55.1)	34 (44.2)	77 (49.7)
≥ 4	35 (44.9)	43 (55.8)	78 (50.3)
Mean ± standard deviation	3.3 ± 1.2	3.5 ± 1.1	3.4 ± 1.2
Median (range)	3.0 (0–5)	4.0 (1–5)	4.0 (0–5)
Test method used at the study site to measure COVID-19 viral load			
RT-PCR	71 (91.0)	71 (92.2)	142 (91.6)
LAMP test	7 (9.0)	5 (6.5)	12 (7.7)
Missing	0	1 (1.3)	1 (0.6)
Sample type used for negative/positive determination			
Nasopharyngeal swab	56 (71.8)	52 (67.5)	108 (69.7)
Nasal swab	9 (11.5)	7 (9.1)	16 (10.3)
Saliva	13 (16.7)	17 (22.1)	30 (19.4)
Missing	0	1 (1.3)	1 (0.6)
SARS-CoV-2 viral load (central laboratory) (log_10_ copies/mL)			
< 7	39 (50.0)	45 (58.4)	84 (54.2)
≥ 7	39 (50.0)	32 (41.6)	71 (45.8)
Mean ± standard deviation	6.69 ± 1.48	6.41 ± 1.69	6.55 ± 1.59
Median (range)	6.98 (3.40–9.08)	6.61 (3.40–9.40)	6.91 (3.40–9.40)
Ordinal scale for severity			
3: Hospitalized, no oxygen therapy	78 (100.0)	77 (100.0)	155 (100.0)
Presence of lung lesions	38 (48.7)	42 (54.5)	80 (51.6)
IgM antibody test (central laboratory)			
Positive	1 (1.3)	2 (2.6)	3 (1.9)
Negative	77 (98.7)	75 (97.4)	152 (98.1)
Mean ± standard deviation	0.023 ± 0.081	0.042 ± 0.180	0.033 ± 0.139
Median (range)	0.010 (0.00–0.72)	0.010 (0.00–1.14)	0.010 (0.00–1.14)
IgG antibody test (central laboratory)			
Positive	2 (2.6)	0	2 (1.3)
Negative	76 (97.4)	77 (100.0)	153 (98.7)
Mean ± standard deviation	0.078 ± 0.470	0.011 ± 0.009	0.045 ± 0.334
Median (range)	0.010 (0.00–3.91)	0.010 (0.00–0.08)	0.010 (0.00–3.91)
Presence of clinical symptoms	55 (70.5)	53 (68.8)	108 (69.7)

Values are *n* (%) unless otherwise stated

*BMI* body mass index, *RT-PCR* reverse transcriptase-polymerase chain reaction, *LAMP* loop-mediated isothermal amplification, *Ig* immunoglobulin

^a^Includes the following: chronic respiratory disease, chronic kidney disease, diabetes mellitus, hypertension, cardiovascular disease, and obesity (BMI ≥ 30 kg/m^2^)

^b^Duration from COVID-19 symptoms onset date (for asymptomatic patients, the collection date of the sample with positive confirmation) to the registration date

During the treatment period, 134 of 155 patients discontinued treatment/dropped out or terminated treatment early after achieving the study endpoint (two consecutive negative SARS-CoV-2 tests): 68 of 78 (87.2%) in the camostat mesilate group and 66 of 77 (85.7%) in the placebo group (Additional file 2: Table S6). The most frequent reason for discontinuation of treatment was two consecutive negative SARS-CoV-2 tests in accordance with the study protocol in the camostat mesilate (45 patients, 57.7%) and placebo (43 patients, 55.8%) groups. Seven of 78 (9.0%) patients in the camostat mesilate group and six of 77 (7.8%) in the placebo group withdrew at the patient’s request.

### Time to SARS-CoV-2-negative test

**Fig. 2:**
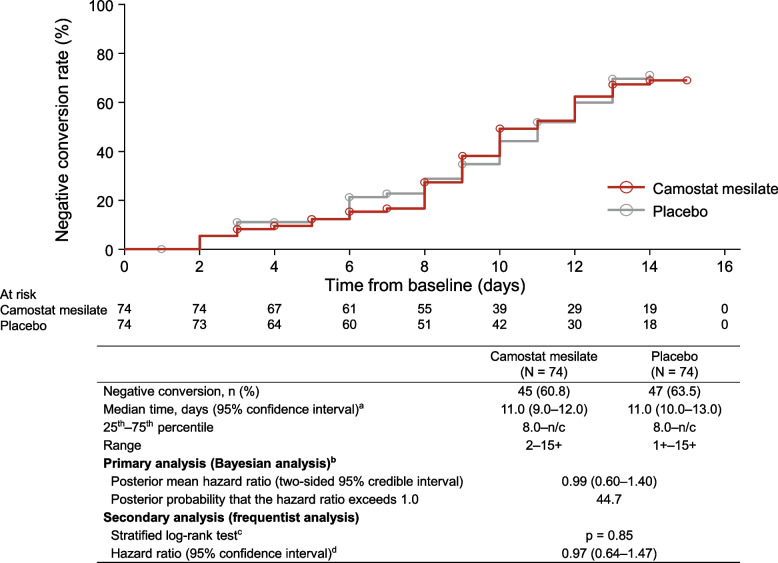
Time to SARS-CoV-2 negative conversion (local laboratory tests). ^a^ The median time to negative conversion was estimated using the Kaplan–Meier method, and the confidence intervals were calculated using the Brookmeyer–Crowley method with double log transformation. ^b^ A Cox proportional hazards model stratified by the randomization factors (age group and underlying diseases) was used to determine the posterior mean hazard ratio with two-sided 95% credible intervals for the camostat mesilate group relative to the placebo group. ^c^ Stratified log-rank test with randomization factors (age group and underlying diseases) as stratification factors. ^d^ Cox proportional hazards model with randomization factors (age group and underlying diseases) as stratification factors and treatment group as the covariate. Age groups: ≥ 65 years *vs* < 65 years. Underlying diseases: chronic respiratory disease, chronic kidney disease, diabetes mellitus, hypertension, cardiovascular disease, and obesity (body mass index ≥ 30 kg/m^2^). *n/c* not calculable

The median time to the first two consecutive SARS-CoV-2 negative tests (local laboratory) was 11 days in both groups (Fig. 2), with conversion to negative status by day 14 in 45 of 74 patients (60.8%) in the camostat mesilate group and 47 of 74 patients (63.5%) in the placebo group. The primary (Bayesian) and secondary (frequentist) analyses confirmed there was no significant difference in the primary endpoint between the two groups. Similar results were obtained in the results of the sensitivity analysis and central laboratory tests (Additional file 2: Tables S7 and S8). Subgroup analyses were also conducted to evaluate the potential influence of patient characteristics, such as underlying diseases and antibodies. However, there were no differences in the efficacy of camostat mesilate or placebo in terms of the time to a negative SARS-CoV-2 test among any of the subgroups evaluated, including the baseline viral load (Additional file 2: Table S9).

### Viral load

**Fig. 3:**
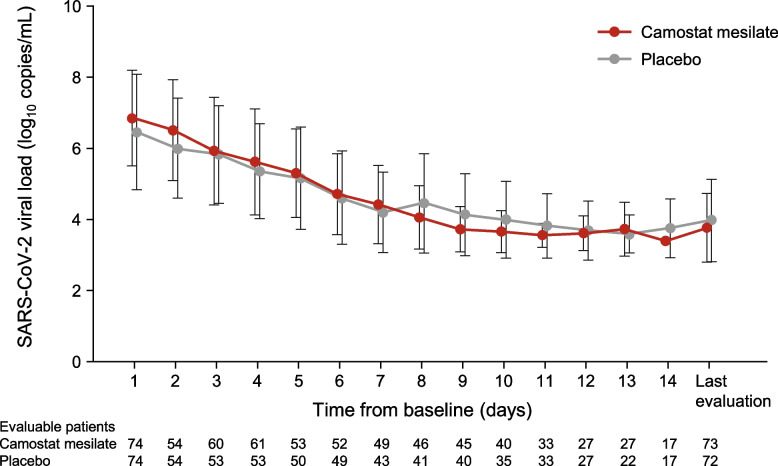
Change in SARS-CoV-2 viral load over time. Values are mean ± standard deviation

The viral load was monitored daily in all patients. As illustrated in Fig. 3, the changes in viral load over time were comparable in both groups, and there were no apparent differences at any time point.

### Ordinal scale of severity

**Fig. 4:**
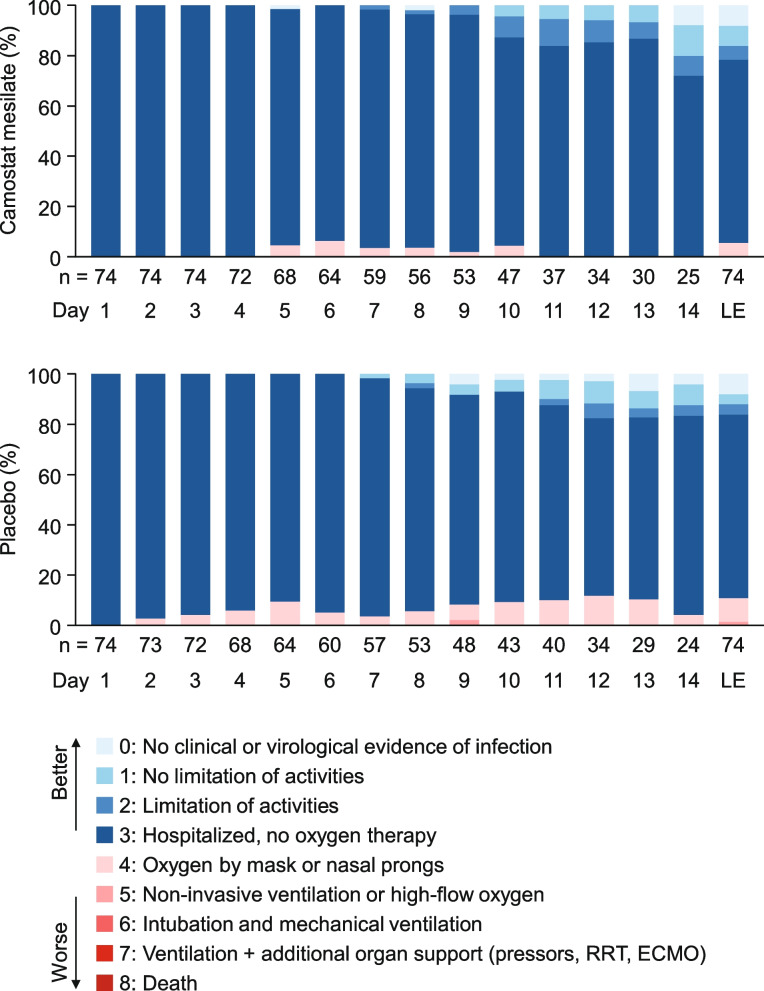
Ordinal scale for severity. The vertical axes show the cumulative percentages of patients. *LE* last evaluation *RRT* renal replacement therapy, *ECMO* extracorporeal membrane oxygenation

The distribution of the ordinal scale of severity was comparable in both groups, with no clear differences at any time (Fig. 4). The ordinal scale was grade 3 (hospitalized, no oxygen therapy) in most patients during the study period because all patients were hospitalized for SARS-CoV-2 testing; outpatients were not enrolled due to the risk of transmission. Nevertheless, none of the patients in either group required intubation/mechanical ventilation or ventilation plus additional organ support, and there were no deaths. The most severe case was a patient in the placebo group whose severity was classified as grade 5 (requiring non-invasive ventilation or high-flow oxygen therapy) on day 9. Other than the patient classified as grade 5 on day 9, none of the other patients in either group experienced a worsening in the ordinal scale by at least two categories at any time during the study.

### Resolution of clinical symptoms

The median time to resolution of clinical symptoms was 13 days in the camostat mesilate and 12 days in the placebo group (Table 2).

**Table 2:** Time to resolution of clinical symptoms

	**Camostat mesilate (*N* = 53)**	**Placebo (*N* = 52)**
Events, *n* (%)	23 (43.4)	23 (44.2)
Median (95% CI) (days)	13.0 (10.0–n/c)	12.0 (10.0–n/c)
25th to 75th percentile	7.0–15.0	9.0–n/c
Range	2–16 +	2–14 +

+ censored, *CI* confidence interval, *n/c* not calculable

### Safety

Adverse events occurred in 25 of 77 patients in the camostat mesilate group (32.5%) and in 31 of 76 patients in the placebo group (40.8%) (Table 3). A serious adverse event (prinzmetal angina) occurred in one patient in the camostat mesilate group, but it was not considered related to the study drug. Adverse drug reactions were reported in nine patients in the camostat mesilate group and in seven patients in the placebo group. Two patients in the camostat mesilate group discontinued treatment due to adverse drug reactions (hepatic function abnormal and drug eruption). The most common types of adverse drug reactions in the camostat mesilate group were gastrointestinal disorders (Table 3).

**Table 3:** Safety data

	**Camostat mesilate (*N* = 77)**	**Placebo (*N* = 76)**	**Total (*N* = 153)**
Number of patients with			
Any adverse events	25 (32.5)	31 (40.8)	56 (36.6)
Any serious adverse events	1 (1.3)	0	1 (0.7)
Any adverse events that led to discontinuation of treatment	2 (2.6)	0	2 (1.3)
Any adverse drug reactions	9 (11.7)	7 (9.2)	16 (10.5)
Serious adverse drug reactions	0	0	0
Adverse drug reactions that led to discontinuation of treatment	2 (2.6)	0	2 (1.3)
Adverse events or adverse drug reactions resulting in death	0	0	0
Adverse drug reactions by system organ class/preferred term			
Gastrointestinal disorders	4 (5.2)	5 (6.6)	9 (5.9)
Abdominal discomfort	1 (1.3)	1 (1.3)	2 (1.3)
Constipation	1 (1.3)	0	1 (0.7)
Diarrhea	2 (2.6)	1 (1.3)	3 (2.0)
Nausea	0	1 (1.3)	1 (0.7)
Stomatitis	0	1 (1.3)	1 (0.7)
Vomiting	0	1 (1.3)	1 (0.7)
Hepatobiliary disorders	1 (1.3)	1 (1.3)	2 (1.3)
Hepatic function abnormal	1 (1.3)	1 (1.3)	2 (1.3)
Laboratory tests	3 (3.9)	2 (2.6)	5 (3.3)
Alanine aminotransferase increased	1 (1.3)	2 (2.6)	3 (2.0)
Aspartate aminotransferase increased	0	1 (1.3)	1 (0.7)
Blood potassium increased	2 (2.6)	0	2 (1.3)
Blood alkaline phosphatase increased	0	1 (1.3)	1 (0.7)
Skin and subcutaneous tissue disorders	1 (1.3)	1 (1.3)	2 (1.3)
Drug eruption	1 (1.3)	0	1 (0.7)
Rash papular	0	1 (1.3)	1 (0.7)

Values are *n* (%)

## Discussion

SARS-CoV-2 remains a clinically significant global health crisis and there remains an urgent, ongoing need to identify effective treatments. A number of preclinical studies have been conducted in the search for therapies for COVID-19. It was discovered that GBPA, the active metabolite of camostat mesilate, inhibits TMPRSS2 and prevents SARS-CoV-2 infection of human airway cells [7–14]. Furthermore, a small-scale retrospective study suggested a potential therapeutic effect of camostat mesilate in patients admitted to an intensive care unit [22].

Despite promising results of preclinical studies, as well as a small retrospective study, the results of our study indicate that camostat mesilate did not substantially reduce the time to viral clearance compared with placebo for treating patients with mild to moderate SARS-CoV-2 infection with or without symptoms. The lack of antiviral effect was demonstrated based on the median time to the first two consecutive SARS-CoV-2-negative tests (the primary endpoint) and the other clinical outcomes, with no statistically significant or clinically relevant differences between the two groups. Although this study investigated a high dose of camostat mesilate (600 mg qid; four to eight times higher than the clinical doses in Japan), no new safety concerns were identified.

Another large-scale study conducted in Denmark and Sweden also reported no benefit of administering camostat mesilate at a dose of 200 mg three times daily (lower than the dose used in our study, 600 mg qid) or placebo for 5 days in hospitalized patients [23]. The primary endpoint in that study was a composite of the time to discharge or a clinical improvement in clinical severity of at least 2 points on a 7-point ordinal scale. The median time to clinical improvement was 5 days in both groups.

Additionally, a US study of outpatients treated with camostat mesilate at a dose of 200 mg four times daily for 7 days did not achieve a reduction in viral load compared with placebo [24]. Interestingly, the efficacy of camostat mesilate was not confirmed by the primary endpoint of viral load, but camostat mesilate prevented the loss of smell/taste compared with placebo. Alterations in smell and taste are common in patients with COVID-19 and remain an unmet medical need. Therefore, these results are noteworthy even with the small sample size of 70 patients.

From the data available to date, it is unclear why the findings of preclinical studies did not translate into a clinical effect. However, several factors related to the design of the study and the mechanism of action of camostat mesilate should be considered:

- *Viral entry pathway*—Although TMPRSS2 is one of the primary routes of viral entry, the viral particles might exploit other pathways, such as endocytosis, to compensate for reduced entry via TMPRSS2. Thus, effective treatments may require inhibition of multiple viral entry pathways, and a combination of drugs with various mechanisms of action, including camostat mesilate, may be useful for treating COVID-19 [25].
- *Inappropriate timing of administration*—The peak viral load is typically reached within 2–3 days after the onset of symptoms, and the administration of camostat mesilate was started approximately 3 days after the onset of symptoms in this study. The selected timing of administration of camostat mesilate might not have been best optimized to suppress viral activity. It has been suggested that therapies aimed at blocking infection or viral reproduction may be more effective if they are initiated before the peak viral load [26]. Therefore, some efficacy may be observed if the administration of camostat mesilate is started as early as possible in the course of infection, perhaps as prophylactic administration to close contacts of patients, such as household members, or immediately after a positive test result. In fact, antibody drugs such as casirivimab/imdevimab have been shown to reduce the risk of onset in uninfected patients and to prevent aggravation [27].
- *Dosing*—Prior to this study, we conducted a phase 1 study to set the dosage and treatment regimen for this study. An important pharmacokinetic (PK) feature of camostat mesilate is that when orally administered, it is rapidly metabolized into an active metabolite (GBPA) by esterases [28–30]. Those studies demonstrated that camostat mesilate is not detectable in human plasma and that GBPA is rapidly eliminated with a half-life of less than 2 h. Therefore, frequent dosing is required to maintain target plasma concentrations. Considering the adherence of the target patient population, we assumed that qid administration of camostat mesilate (morning, midday, evening, and before bedtime) would be acceptable. Specifically, in PK/pharmacodynamic (PD) simulations, the times above the EC_50_ of camostat mesilate at doses of 800 mg three times daily and 600 mg qid were 9.8 h and 11.5 h, respectively [20]. The results of the PK/PD simulations also suggested an advantage of increasing the dosing frequency rather than increasing the dose per administration. Multiple administrations of camostat mesilate at 600 mg qid were well tolerated in a phase 1 study. However, in a repeated-dose toxicity study in dogs, camostat 300 mg/kg decreased body weight and food intake; induced vomiting and effects on the gastrointestinal tract, including gastrointestinal injury; and caused death, with a no-observed adverse effect level (NOAEL) of 100 mg/kg [20, 31]. Converting the NOAEL in dogs to humans yielded an equivalent dose of 3333 mg [20, 32]. Thus, the dose used in this study had a safety margin of 1.4-fold. Based on the overall balance between the expected time above EC_50_ and safety risks, 600 mg qid was determined to be an appropriate dose for this study. The plasma concentration of GBPA was predicted to exceed the EC_50_ for at least 11.5 h at the dose used (2400 mg/day) [20]. However, the targeted time above EC_50_ might have been insufficient to inhibit TMPRSS2 and hence prevent viral entry, although the exact relationship between the exposure and antiviral activity is not clear in the clinic.

A limitation of this study is that the improvement of the ordinal scale of severity could not be evaluated correctly because most patients were hospitalized for daily viral testing regardless of the presence or absence of symptoms and were hence classified as grade 3. Another possible limitation is that the effects of camostat mesilate against SARS-CoV-2 were evaluated using nasopharyngeal and nasal swab samples in the majority of patients. However, the appropriateness of an index of upper airway viral load in asymptomatic to moderate cases remains questionable. It is considered that the epidemic strain at the time was a D614G strain, but no data on the type of strain were collected for this study. Efficacy against currently circulating variants is unknown. In addition, the antiviral activity was assessed as the time to negative SARS-CoV-2 tests, but this endpoint is dependent on the baseline viral load, the assay used, and has been shown to have a “tail” with low viral loads persisting over time [33]. These factors might have influenced the results of the virological endpoints used in this study.

There are some strengths of this study that should be mentioned. In particular, this was a double-blind, randomized, placebo-controlled study with robust randomization as demonstrated by the high similarity of both groups. In addition, this study used a dose that was four to eight times higher than the clinical doses in Japan used for the acute symptoms of chronic pancreatitis and postoperative reflux esophagitis based on the preclinical and early clinical evidence. Furthermore, the efficacy of camostat mesilate was assessed using multiple clinically relevant endpoints, including local and central laboratory tests for SARS-CoV-2 infection and viral load.

Although the study results were negative, there were several lessons, and the study generated important new evidence. There are still some questions related to the development of clinical trials for emerging infectious diseases, including study design and patient segmentation. Even in a state of emergency, the doses in clinical trials should be carefully selected with consideration of clinical pharmacology, including PK/PD modeling and simulation, when planning clinical trials for a new drug candidate in settings such as this, in order to provide clear evidence supporting or halting ongoing development of the drug. Furthermore, a collaboration between government, industry, and academia is essential for the development of therapeutic agents in a pandemic.

## Conclusions

In conclusion, the results of this study found clear evidence for not using camostat mesilate to treat mild to moderate SARS-CoV-2 infection with or without symptoms. Of note, no new safety concerns were identified at the high dose used in this study, which exceeds the standard dose used in other indications. Overall, these findings highlight the continuing need to identify and develop alternative therapies for COVID-19 and the necessity of conducting well-designed studies to confirm whether preclinical findings translate into meaningful clinical efficacy.

## Supplementary Information

**Additional file 1:** Study protocol.

**Additional file 2:** IRB information. Sample size calculation. **Table S1.** Discontinuation criteria. **Table S2.** Ordinal scale of severity. **Table S3.** Events and reasons for censoring patients for the analysis of time to SARS-CoV-2 negativity. **Table S4.** Events and reasons for censoring patients in the sensitivity analysis of the time to SARS-CoV-2 negativity. **Table S5.** Changes to the statistical analyses plan from the protocol. **Table S6.** Patient disposition. **Table S7.** Results of the sensitivity analysis of the primary endpoint. **Table S8.** Time to negative SARS-CoV-2 status as measured by the central laboratory. **Table S9.** Results of the subgroup analyses of time to negative SARS-CoV-2 status.

**Additional file 3:** CONSORT Checklist.

## Data Availability

The datasets generated during and/or analyzed during the current study are not publicly available to protect the patients’ privacy. However, researchers may use the study protocol (Additional file 1: Study protocol) and all data published in this manuscript and its associated files. Furthermore, qualified researchers may request Ono Pharma to disclose individual patient-level data from clinical studies through the following website: https://www.clinicalstudydatarequest.com/. For more information on Ono Pharma’s Policy for the Disclosure of Clinical Study Data, please see the following website: https://www.ono.co.jp/eng/rd/policy.html.

## Abbreviations

ACE2: Angiotensin-converting enzyme II
BMI: Body mass index
CANDLE: Camostat could bring delight to people
EC_50_: Half maximal effective concentration
GBPA: 4-(4-Guanidinobenzoyloxy)phenylacetic acid
LAMP: Loop-mediated isothermal amplification
NOAEL: No-observed adverse effect level
PD: Pharmacodynamic
PK: Pharmacokinetic
RT-PCR: Reverse transcriptase-polymerase chain reaction
TMPRSS2: Type II transmembrane serine protease
