# Supplementary material for: A multicenter, double-blind, randomized, parallel-group, placebo-controlled study to evaluate the efficacy and safety of camostat mesilate in patients with COVID-19 (CANDLE study)
Source: BMC Med. 2022 Sep 27;20:342. doi: 10.1186/s12916-022-02518-7 (PMC9512971; doi:10.1186/s12916-022-02518-7)
Supplement: Supplementary file 2 — Additional file 2: IRB information. Sample size calculation. Table S1. Discontinuation criteria. Table S2. Ordinal scale of severity. Table S3. Events and reasons for censoring patients for the analysis of time to SARS-CoV-2 negativity. Table S4. Events and reasons for censoring patients in the sensitivity analysis of the time to SARS-CoV-2 negativity. Table S5. Changes to the statistical analyses plan from the protocol. Table S6. Patient disposition. Table S7. Results of the sensitivity analysis of the primary endpoint. Table S8. Time to negative SARS-CoV-2 status as measured by the central laboratory. Table S9. Results of the subgroup analyses of time to negative SARS-CoV-2 status. [file 12916_2022_2518_MOESM2_ESM.pdf]

SUPPLEMENTARY INFORMATION

A multicenter, double-blind, randomized, parallel-group, placebo-controlled study to evaluate the efficacy and safety of camostat mesilate in patients with COVID-19 (CANDLE study)

Taku Kinoshita, Masahiro Shinoda, Yasuhiro Nishizaki, Katsuya Shiraki, Yuji Hirai, Yoshiko Kichikawa, Kenji Tsushima, Masaharu Sinkai, Naoyuki Komura, Kazuo Yoshida, Yasutoshi Kido, Hiroshi Kakeya, Naoto Uemura, Junichi Kadota

Contents

	Page
IRB information	2
Sample size calculation	3
Table S1. Discontinuation criteria	6
Table S2. Ordinal scale of severity	7
Table S3. Events and reasons for censoring patients for the analysis of time to SARS-CoV-2 negativity	8
Table S4. Events and reasons for censoring patients in the sensitivity analysis of the time to SARS-CoV-2 negativity	9
Table S5. Changes to the statistical analyses plan from the protocol	10
Table S6. Patient disposition	12
Table S7. Results of the sensitivity analysis of the primary endpoint	13
Table S8. Time to negative SARS-CoV-2 status as measured by the central laboratory	15
Table S9. Results of the subgroup analyses of time to negative SARS-CoV-2 status	16

IRB information

The protocol and patient consent forms were reviewed and approved by the following institutional review boards or ethics committees at each participating center: Haradoi Hospital Institutional Review Board, Higashiosaka City Medical Center Institutional Review Board, Institutional Review Board of Tokyo Medical University Hachioji Medical Center, International University of Health and Welfare Institutional Review Board, Juntendo University Hospital Institutional Review Board, Kantorosai Hospital Institutional Review Board, Kobori Central Clinical Research Ethics Committee, Koseiren Hospital Central Institutional Review Board, Mishuku Hospital Institutional Review Board, National Hospital Organization Kanazawa Medical Center Institutional Review Board, Nihon University Hospitals' Joint Institutional Review Board, Okayama City Hospital Institutional Review Board, Review Board of Human Rights and Ethics for Clinical Studies Institutional Review Board, Saiseikai Kurihashi Hospital Institutional Review Board, Saitama Medical Center Institutional Review Board, Saitama Prefectural Cardiovascular and Respiratory Center Institutional Review Board, Tachikawa Hospital Institutional Review Board, Tokai University Hospital Group Institutional Review Board, Tokushukai Group Institutional Review Board, Tokyo Medical and Dental University Hospital Institutional Review Board, Tokyo Metropolitan Hiroo Hospital Institutional Review Board, Tokyo Shinagawa Hospital Institutional Review Board, Yokohama Municipal Citizen's Hospital Institutional Review Board, and Yokosuka Kyosai Hospital Institutional Review Board.

Sample size calculation

The objective of this study was to establish superiority of camostat mesilate over placebo in patients with COVID-19 using the time to negative SARS-CoV-2 test as the primary endpoint. Patients were to be randomized 1:1 to either camostat mesilate or placebo using a dynamic allocation procedure with site, age (≥65 *vs* <65 years), and presence/absence of underlying diseases (chronic respiratory disease, chronic kidney disease, diabetes mellitus, hypertension, cardiovascular diseases, obesity [body mass index ≥30 kg/m^2]) as allocation factors.

If enrolment became difficult due to convergence of COVID-19, an interim analysis was planned to assess the effectiveness or ineffectiveness at the time by applying Bayesian interim monitoring[1] with the criteria for assessment in the interim and final analyses (**Table A**). The criteria for the assessment in the final analysis were kept regardless of whether an interim analysis was performed.

Table A. Criteria for assessing effectiveness/ineffectiveness using the Bayesian posterior probability method

Time of analysis	Criteria for effectiveness	Criteria for ineffectiveness
Interim analysis	Bayesian posterior probability ≥97.5% for hazard ratio >1.0	Bayesian posterior probability ≤2.5% for hazard ratio >1.0 AND Bayesian posterior probability ≥80.0% for hazard ratio <1.4
Final analysis	Bayesian posterior probability ≥92% for hazard ratio >1.0	Bayesian posterior probability ≤8% for hazard ratio >1.0

The time to negative SARS-CoV-2 test was assumed to follow an exponential distribution, with a median time of 14 days in the placebo group and a median time of 7–8 days in the camostat mesilate group. The probabilities of meeting the assessment of effectiveness or ineffectiveness with 50 subjects per group (100 in total) were calculated at various analysis time-points by applying numerical simulations in SAS version 9.4. Because the timing of the interim analysis was dependent on the status of the COVID-19 outbreak, numerical simulations were performed, assuming the time-points when 40 to 80 subjects would have been randomized. The prior distribution of the regression coefficient was assumed to be uniform. The numerical experiments were repeated 1,000 times under these conditions, and the probabilities of meeting the assessment of effectiveness and ineffectiveness at various time-points were estimated as shown in **Table B**.

Table B. Simulated effectiveness and ineffectiveness rates according to predicted time to negative SARS-CoV-2 and number of patients

Median time (days) in the camostat mesilate group	Number of patients at the time of the interim analysis	Assessment	Interim analysis (%)	Final analysis (%)	Total (%)
7	40	Effective	36.6	52.2	88.8

Median time (days) in the camostat mesilate group	Number of patients at the time of the interim analysis	Assessment	Interim analysis (%)	Final analysis (%)	Total (%)
		Ineffective	4.3	6.9	11.2
	50	Effective	44.1	46.2	90.3
		Ineffective	2.4	7.3	9.7
	60	Effective	51.1	39.1	90.2
		Ineffective	2.2	7.6	9.8
	70	Effective	57.5	32.5	90.0
		Ineffective	1.5	8.5	10.0
	80	Effective	64.3	25.9	90.2
		Ineffective	1.5	8.3	9.8
8	40	Effective	23.1	52.2	75.3
		Ineffective	7.6	17.1	24.7
	50	Effective	29.8	45.5	75.3
		Ineffective	6.5	18.2	24.7
	60	Effective	36.1	40.6	76.7
		Ineffective	6.1	17.2	23.3
	70	Effective	39.1	38.0	77.1
		Ineffective	4.6	18.3	22.9
	80	Effective	45.5	31.1	76.6
		Ineffective	4.9	18.5	23.4
14	40	Effective	2.4	6.9	9.3
		Ineffective	43.6	47.1	90.7
	50	Effective	2.6	6.6	9.2
		Ineffective	47.2	43.6	90.8
	60	Effective	2.8	6.4	9.2
		Ineffective	49.2	41.6	90.8
	70	Effective	2.2	6.8	9.0
		Ineffective	53.7	37.3	91.0
	80	Effective	2.3	6.2	8.5
		Ineffective	56.0	35.5	91.5

Based on the numerical experiment, irrespective of which time-point the interim analysis was performed, the probability that treatment would be effective was 75% to 90% if the median duration was 7 or 8 days in the camostat mesilate group. Moreover, if the median duration was 14 days, the probability that treatment was effective was controlled within 10%. From these data, we therefore considered it was possible to demonstrate superiority of

camostat mesilate over placebo with a sample size of 100 patients (50 patients per group).

Reference

1. Teramukai S. Bayesian design of single-arm clinical trials with binary endpoints: a review. Jpn J Biometrics. 2008;29:111–24 [In Japanese].

Table S1. Discontinuation criteria

**No.**	**Criterion**
1	The patient requested to withdraw from the study
2	The patient's representative requested to withdraw the patient from the study
3	The patient did not meet the inclusion criteria or met the exclusion criteria
4	Continuation of the study was considered difficult because of adverse event(s), whether or not related to the study drug, in the opinion of the investigator or subinvestigator
5	The patient tested negative for SARS-CoV-2 on two consecutive occasions
6	If criteria *a* and *b* are both met, and continuation of the study was considered difficult:<br>a. Exacerbation of pneumonia was observed by chest imaging<br>b. Oxygen saturation was ≤93% despite oxygen therapy
7	The patient received a prohibited concomitant medication
8	The patient died
9	The patient was found to be pregnant
10	Continuation of the study was not appropriate for any other reason(s), in the opinion of the investigator or subinvestigator

Table S2. Ordinal scale of severity [1]

**Patient state**	**Description**	**Grade**
Uninfected	- No clinical or virological evidence of infection	0
Ambulatory	- No limitation of activities	1
	- Limitations of activities	2
Hospitalized		
Mild disease	- Hospitalized, no oxygen therapy	3
	- Oxygen therapy with a mask or nasal prongs	4
Severe disease	- Non-invasive ventilation or high-flow oxygen therapy	5
	- Intubation and mechanical ventilation	6
	- Ventilation and additional organ support (e.g., pressors, renal replacement therapy, extracorporeal membrane oxygenation)	7
Dead	- Death	8

1 World Health Organization R&D Blue Print Team. WHO R&D Blueprint: novel coronavirus - COVID-19 therapeutic trial synopsis. Available at: https://www.who.int/publications/i/item/covid-19-therapeutic-trial-synopsis. Accessed: July 22, 2021.

Table S3. Events and reasons for censoring patients for the analysis of time to SARS-CoV-2 negativity

**Status**	**Event or censored date**	**Result**
SARS-CoV-2 negativity was determined	Sampling date of the sample tested negative for the first time for the SARS-CoV-2 test (measured at the study site)	Event
SARS-CoV-2 negativity was not determined	Sampling date of the last assessable SARS-CoV-2 test (measured at the study site)	Censored
Died	Day 14	Censored
The patient met criteria for discontinuing the study drug (study drug discontinuation criteria 6 or 7) for worsening of COVID-19, and administration of the study drug was discontinued	Day 14	Censored
Study was being discontinued	Sampling date of the last assessable SARS-CoV-2 test (measured at the study site)	Censored
SARS-CoV-2 test (measured at the study site) was not performed more than once after randomization	Day 1	Censored

Table S4. Events and reasons for censoring patients in the sensitivity analysis of the time to SARS-CoV-2 negativity

Status	Event or censored date	Result
Patient tested negative for SARS-CoV-2, and did not test positive for SARS-CoV-2 thereafter	Sampling date of the sample tested negative for the first time for the SARS-CoV-2 test (measured at the study site)	Event
There were several determinations of SARS-CoV-2 negativity	Sampling date of the sample tested negative for the first time for the SARS-CoV-2 test at the latest determination (measured at the study site)	Event
Patient tested positive for SARS-CoV-2 after a negative test for SARS-CoV-2	Sampling date of the last assessable SARS-CoV-2 test (measured at the study site)	Censored
SARS-CoV-2 negativity was not determined	Sampling date of the last assessable SARS-CoV-2 test (measured at the study site)	Censored
Died	Day 14	Censored
The patient met criteria for discontinuing the study drug (study drug discontinuation criteria 6 or 7) for worsening of COVID-19, and administration of the study drug was discontinued	Day 14	Censored
Study was being discontinued	Sampling date of the last assessable SARS-CoV-2 test (measured at the study site)	Censored
SARS-CoV-2 test (measured at the study site) was not performed more than once after randomization	Day 1	Censored

Table S5. Changes to the statistical analyses plan from the protocol

**Description in the protocol**	**Description in the statistical analysis plan**	**Reason for change**
None	9 Primary efficacy endpoint<br>9.3 Analysis methods<br>Addition of sensitivity analysis	To investigate the effect of patients who tested positive for SARS-CoV-2 test after their SARS-CoV-2 test became negative
10.5.5 Secondary efficacy endpoint<br>10.5.5.2.1 Analysis item<br>Percentage of patients with a negative SARS-CoV-2 test	10 Secondary efficacy endpoints<br>10.2 Analysis items and data handling<br>Cumulative proportion of patients with a negative SARS-CoV-2 test	Based on the definition of the determination of negative conversion and the status of data collection after the determination of negative conversion.<br>To evaluate effectiveness in more detail
None	10 Secondary efficacy endpoints<br>10.3 Analysis methods<br>Addition of summary statistics to the ordinal scale of severity	To evaluate effectiveness in more detail
10.5.5 Secondary efficacy endpoint<br>10.5.5.3 Analysis method<br>Analysis method for survival	10 Secondary efficacy endpoints<br>10.3 Analysis methods<br>Only events, number of censored patients, reasons for censoring, and Kaplan–Meier curves were analyzed for survival	Data on survival status were confirmed in a blinded manner, and detailed analysis was considered unnecessary
None	11 Exploratory efficacy endpoints<br>11.2 Analysis items and data handling<br>Time to onset of clinical symptoms and addition of the proportion of patients with clinical symptoms	To evaluate effectiveness in more detail

Description in the protocol	Description in the statistical analysis plan	Reason for change
None	11 Exploratory efficacy endpoints<br>11.3 Analysis methods<br>The addition of an analysis of the difference in the proportion between the treatment groups in the presence or absence of lung lesions in chest images, the proportion of patients in whom clinical symptoms disappeared, and the proportion of patients in whom clinical symptoms occurred	To evaluate effectiveness in more detail
8.2.5.2 Laboratory tests<br>Hematological examination	12.2 Analysis items and data handling<br>1) Analysis item<br>2) Laboratory test: hematological examination<br>Add total neutrophil count	Because some facilities could not measure the segmented and stab cells separately, an additional item was added to sum the segmented and stab cells

Table S6. Patient disposition

	Camostat mesilate (N = 78)	Placebo (N = 77)	Total (N = 155)
At the end of the observation period			
Number of patients who discontinued or dropped out	0	0	0
At the end of the treatment period			
Number of patients who discontinued or dropped out	68 (87.2)	66 (85.7)	134 (86.5)
Reason for discontinuation or dropout			
Withdrawn at the patient's request	7 (9.0)	6 (7.8)	13 (8.4)
Withdrawn at the guardian's request	0	0	0
Adverse event	2 (2.6)	0	2 (1.3)
Physician's decision	9 (11.5)	11 (14.3)	20 (12.9)
Protocol deviation	0	0	0
Pregnancy	0	0	0
Lost to follow-up	0	0	0
Non-compliance with study drug	0	0	0
Study terminated by sponsor	0	0	0
Death	0	0	0
Protocol-specified withdrawal criterion met	47 (60.3)	48 (62.3)	95 (61.3)
Discontinuation criterion 5	45 (57.7)	43 (55.8)	88 (56.8)
Discontinuation criterion 6	2 (2.6)	5 (6.5)	7 (4.5)
Other	3 (3.8)	1 (1.3)	4 (2.6)
At the end of the study			
Number of patients who discontinued or dropped out	3 (3.8)	5 (6.5)	8 (5.2)
Reason for discontinuation or dropout			
Withdrawn at the patient's request	1 (1.3)	1 (1.3)	2 (1.3)
Withdrawn at the guardian's request	0	0	0
Adverse event	0	0	0
Death	0	1 (1.3)	1 (0.6)
Pregnancy	0	0	0
Other	2 (2.6)	3 (3.9)	5 (3.2)

Values are n (%)

Table S7. Results of the sensitivity analysis of the primary endpoint

	Camostat mesilate (N = 74)	Placebo (N = 74)
Negative conversion, n (%)	42 (56.8)	45 (60.8)
Number of censored patients, n (%)	32 (43.2)	29 (39.2)
Reasons for censoring patients, n (%)		
The patient tested positive for SARS-CoV-2 after a negative test for SARS-CoV-2	3 (9.4)	2 (6.9)
SARS-CoV-2 negativity was not confirmed	27 (84.4)	21 (72.4)
Death	0	1 (3.4)
The patient met criteria for discontinuing the study drug (study drug discontinuation criteria 6 or 7) for worsening of COVID-19, and administration of the study drug was discontinued	2 (6.3)	4 (13.8)
Patient discontinued the study	0	0
SARS-CoV-2 test (measured at the study site) was not performed more than once after randomization	0	1 (3.4)
Analysis of time (in days) to a negative test		
Median time (95% confidence interval)[a]	12.0 (10.0–13.0)	11.0 (10.0–13.0)
25th to 75th percentile[a]	8.0–n/c	8.0–n/c
Range	2–15+	1+–14+
Main analysis (Bayesian analysis)		
Posterior average of hazard ratio (two-sided 95% credible interval)[b]	0.96 (0.57–1.36)	
Posterior probability that the hazard ratio exceeds 1.0[b]	38.0	
Secondary analysis (frequency analysis)		
Stratified log-rank test[c]	p = 0.71	
Hazard ratio (95% confidence interval)[b]	0.94 (0.61–1.43)	

[a]Estimated using the Kaplan–Meier method. The Brookmeyer-Crowley method with double log transformation was used to calculate the confidence interval

[b]Randomization factors (age [≥65 years *vs* <65 years] and presence of underlying diseases [chronic respiratory disease, chronic kidney disease, diabetes mellitus, hypertension, cardiovascular disease, and obesity (BMI ≥30 kg/m^2)]) were estimated using a stratified Cox proportional hazards model with stratification factors and treatment group as covariates

[c]Stratified log-rank test with randomization factors (age [≥65 years *vs* <65 years] and presence of underlying diseases [chronic respiratory disease, chronic kidney disease, diabetes mellitus, hypertension, cardiovascular disease, obesity (BMI ≥30 kg/m^2)]) as stratification factors

+ censored, *n/c* not calculable

Table S8. Time to negative SARS-CoV-2 status as measured at the central laboratory

	**Camostat mesilate (N = 73)**	**Placebo (N = 72)**
Negative conversion, n (%)	30 (41.1)	30 (41.7)
Number of censored patients, n (%)	43 (58.9)	42 (58.3)
Reasons for censoring patients, n (%)		
SARS-CoV-2 negative status not confirmed	39 (90.7)	33 (78.6)
Death	0	1 (2.4)
The patient met criteria for discontinuing the study drug (study drug discontinuation criteria 6 or 7) for worsening of COVID-19, and administration of the study drug was discontinued	2 (4.7)	3 (7.1)
Patient discontinued the study	0	0
SARS-CoV-2 test (measured at the study site) was not performed more than once after randomization	2 (4.7)	5 (11.9)
Analysis of time (in days) to a negative test		
Median time (95% confidence interval) [a]	12.0 (10.0–n/c)	12.0 (10.0–n/c)
25th to 75th percentile[a]	9.0–n/c	9.0–n/c
Range	1+–15+	1+–14+
Main analysis (Bayesian analysis)		
Posterior average of hazard ratio (two-sided 95% credible interval)[b]	0.86 (0.45–1.31)	
Posterior probability that the hazard ratio exceeds 1.0 [b]	23.8	
Secondary analysis (frequency analysis)		
Stratified log-rank test[c]	p = 0.51	
Hazard ratio (95% confidence interval)[b]	0.83 (0.50–1.39)	

[a]Estimated using the Kaplan–Meier method. The Brookmeyer-Crowley method with double log transformation was used to calculate the confidence interval

[b]Randomization factors (age [≥65 years *vs* <65 years] and presence of underlying diseases [chronic respiratory disease, chronic kidney disease, diabetes mellitus, hypertension, cardiovascular disease, and obesity (BMI ≥30 kg/m^2)]) were estimated using a stratified Cox proportional hazards model with the stratification factors and treatment group as covariates

[c]Stratified log-rank test with randomization factors (age [≥65 years *vs* <65 years] and presence of underlying diseases [chronic respiratory disease, chronic kidney disease, diabetes mellitus, hypertension, cardiovascular disease, and obesity (BMI ≥30 kg/m^2)]) as stratification factors

+ censored, *n/c* not calculable

Table S9. Results of the subgroup analyses of time to negative SARS-CoV-2 status

**Characteristic**			**Camostat mesilate (N = 74)**	**Placebo (N = 74)**
Age (years)	≥65	Number of events	15 (51.7)	15 (53.6)
		Number censored	14 (48.3)	13 (46.4)
		Median (95% CI)[a]	12.0 (9.0–n/c)	13.0 (10.0–n/c)
		Hazard ratio (95% CI)	1.05 (0.51–2.16)	
	<65	Number of events	30 (66.7)	32 (69.6)
		Number censored	15 (33.3)	14 (30.4)
		Median (95% CI)[a]	11.0 (8.0–12.0)	11.0 (8.0–12.0)
		Hazard ratio (95% CI)	0.94 (0.57–1.55)	
Age (years)	≥75	Number of events	7 (53.8)	5 (38.5)
		Number censored	6 (46.2)	8 (61.5)
		Median (95% CI)[a]	10.5 (8.0–n/c)	n/c (9.0–n/c)
		Hazard ratio	2.03 (0.64–6.46)	
	≥65 to <75	Number of events	8 (50.0)	10 (66.7)
		Number censored	8 (50.0)	5 (33.3)
		Median (95% CI)[a]	12.0 (9.0–n/c)	10.0 (8.0–13.0)
		Hazard ratio	0.64 (0.25–1.64)	
	<65	Number of events	30 (66.7)	32 (69.6)
		Number censored	15 (33.3)	14 (30.4)
		Median (95% CI)[a]	11.0 (8.0–12.0)	11.0 (8.0–12.0)
		Hazard ratio (95% CI)	0.94 (0.57–1.55)	
BMI (kg/m^2)	≥30	Number of events	6 (66.7)	4 (66.7)
		Number censored	3 (33.3)	2 (33.3)
		Median (95% CI)[a]	11.0 (2.0–n/c)	13.0 (11.0–n/c)
		Hazard ratio (95% CI)	2.11 (0.55–8.15)	
	<30	Number of events	39 (60.0)	43 (63.2)
		Number censored	26 (40.0)	25 (36.8)
		Median (95% CI)[a]	11.0 (9.0–13.0)	11.0 (9.0–13.0)
		Hazard ratio (95% CI)	0.91 (0.59–1.41)	
Chronic respiratory disease	Yes	Number of events	7 (63.6)	8 (61.5)
		Number censored	4 (36.4)	5 (38.5)
		Median (95% CI)[a]	10.0 (3.0–n/c)	12.0 (7.0–n/c)
		Hazard ratio (95% CI)	1.50 (0.49–4.62)	

Characteristic			Camostat mesilate (N = 74)	Placebo (N = 74)
	No	Number of events	38 (60.3)	39 (63.9)
		Number censored	25 (39.7)	22 (36.1)
		Median (95% CI)[a]	11.0 (9.0–13.0)	11.0 (9.0–13.0)
		Hazard ratio (95% CI)	0.93 (0.59–1.46)	
Chronic kidney disease	Yes	Number of events	4 (80.0)	2 (50.0)
		Number censored	1 (20.0)	2 (50.0)
		Median (95% CI)[a]	9.0 (2.0–12.0)	n/c (9.0–n/c)
		Hazard ratio (95% CI)	71016179.23 (0.00–n/c)	
	No	Number of events	41 (59.4)	45 (64.3)
		Number censored	28 (40.6)	25 (35.7)
		Median (95% CI)[a]	11.0 (10.0–13.0)	11.0 (10.0–12.0)
		Hazard ratio (95% CI)	0.91 (0.59–1.39)	
Diabetes mellitus	Yes	Number of events	10 (71.4)	5 (41.7)
		Number censored	4 (28.6)	7 (58.3)
		Median (95% CI)[a]	9.0 (5.0–10.0)	n/c (8.0–n/c)
		Hazard ratio (95% CI)	3.78 (1.02–14.04)	
	No	Number of events	35 (58.3)	42 (67.7)
		Number censored	25 (41.7)	20 (32.3)
		Median (95% CI)[a]	12.0 (10.0–14.0)	11.0 (10.0–13.0)
		Hazard ratio(95% CI)	0.78 (0.50–1.22)	
Hypertension	Yes	Number of events	14 (60.9)	9 (47.4)
		Number censored	9 (39.1)	10 (52.6)
		Median (95% CI)[a]	11.0 (9.0–n/c)	13.0 (9.0–n/c)
		Hazard ratio (95% CI)	1.16 (0.44–3.07)	
	No	Number of events	31 (60.8)	38 (69.1)
		Number censored	20 (39.2)	17 (30.9)
		Median (95% CI)[a]	11.0 (9.0–13.0)	11.0 (9.0–13.0)
		Hazard ratio (95% CI)	0.87 (0.53–1.42)	
Cardiovascular disease	Yes	Number of events	1 (25.0)	1 (25.0)
		Number censored	3 (75.0)	3 (75.0)
		Median (95% CI)[a]	n/c (12.0–n/c)	n/c (13.0–n/c)
		Hazard ratio (95% CI)	1.00 (1.00–1.00)	
	No	Number of events	44 (62.9)	46 (65.7)

Characteristic			Camostat mesilate (N = 74)	Placebo (N = 74)
		Number censored	26 (37.1)	24 (34.3)
		Median (95% CI)[a]	10.0 (9.0–12.0)	11.0 (10.0–12.0)
		Hazard ratio (95% CI)	0.93 (0.62–1.42)	
Time (days) from sample collection date to confirmation of SARS-CoV-2 positive status in patients who were asymptomatic at the registration date	<4	Number of events	29 (72.5)	15 (46.9)
		Number censored	11 (27.5)	17 (53.1)
		Median (95% CI)[a]	10.0 (9.0–12.0)	13.0 (10.0–n/c)
		Hazard ratio (95% CI)	1.76 (0.93–3.32)	
	≥4	Number of events	16 (47.1)	32 (76.2)
		Number censored	18 (52.9)	10 (23.8)
		Median (95% CI)[a]	13.0 (9.0–n/c)	10.0 (9.0–12.0)
		Hazard ratio (95% CI)	0.56 (0.30–1.02)	
SARS-CoV-2 viral load (central testing) ($\log_{10}$ copies/mL)	<7	Number of events	26 (74.3)	33 (76.7)
		Number censored	9 (25.7)	10 (23.3)
		Median (95% CI)[a]	9.0 (8.0–9.0)	10.0 (8.0–12.0)
		Hazard ratio (95% CI)	1.15 (0.67–1.98)	
	≥7	Number of events	19 (48.7)	14 (45.2)
		Number censored	20 (51.3)	17 (54.8)
		Median (95% CI)[a]	13.0 (12.0–n/c)	13.0 (10.0–n/c)
		Hazard ratio (95% CI)	0.92 (0.46–1.87)	
Presence of pulmonary lesions	Yes	Number of events	23 (63.9)	27 (67.5)
		Number censored	13 (36.1)	13 (32.5)
		Median (95% CI)[a]	10.0 (8.0–12.0)	11.0 (8.0–13.0)
		Hazard ratio (95% CI)	1.05 (0.60–1.85)	
	No	Number of events	22 (57.9)	20 (58.8)
		Number censored	16 (42.1)	14 (41.2)
		Median (95% CI)[a]	12.0 (10.0–14.0)	12.0 (10.0–13.0)
		Hazard ratio (95% CI)	0.86 (0.47–1.59)	
IgM antibody test (central testing)	Positive	Number of events	1 (100.0)	1 (50.0)
		Number censored	0 (0.0)	1 (50.0)
		Median (95% CI)[a]	11.0 (n/c–n/c)	n/c (2.0–n/c)
		Hazard ratio (95% CI)	1.00 (1.00–1.00)	
	Negative	Number of events	44 (60.3)	46 (63.9)
		Number censored	29 (39.7)	26 (36.1)

**Characteristic**			**Camostat mesilate (N = 74)**	**Placebo (N = 74)**
		Median (95% CI)[a]	11.0 (9.0–13.0)	11.0 (10.0–13.0)
		Hazard ratio (95% CI)	0.96 (0.63–1.45)	
IgG antibody test (central testing)	Positive	Number of events	1 (100.0)	0
		Number censored	0	0
		Median (95% CI)[a]	2.0 (n/c–n/c)	n/c (n/c–n/c)
		Hazard ratio (95% CI)	n/c (n/c–n/c)	
	Negative	Number of events	44 (60.3)	47 (63.5)
		Number censored	29 (39.7)	27 (36.5)
		Median (95% CI)[a]	11.0 (9.0–12.0)	11.0 (10.0–13.0)
		Hazard ratio (95% CI)	0.95 (0.63–1.44)	
Presence of clinical symptom(s)	Yes	Number of events	37 (69.8)	36 (69.2)
		Number censored	16 (30.2)	16 (30.8)
		Median (95% CI)[a]	10.0 (9.0–12.0)	11.0 (10.0–13.0)
		Hazard ratio	1.14 (0.72–1.83)	
	No	Number of events	8 (38.1)	11 (50.0)
		Number censored	13 (61.9)	11 (50.0)
		Median (95% CI)[a]	n/c (9.0–n/c)	12.0 (8.0–n/c)
		Hazard ratio (95% CI)	0.37 (0.13–1.03)	

[a]Estimated using the Kaplan–Meier method

CI confidence interval, *n/c* not calculable, *BMI* body mass index, *Ig* immunoglobulin
